# Circular RNA YAP1 acts as the sponge of microRNA‐21‐5p to secure HK‐2 cells from ischaemia/reperfusion‐induced injury

**DOI:** 10.1111/jcmm.15142

**Published:** 2020-03-11

**Authors:** Tao Huang, Yanwei Cao, Hongyang Wang, Qinghai Wang, Jianlei Ji, Xiaoxia Sun, Zhen Dong

**Affiliations:** ^1^ Department of Kidney Transplantation The Affiliated Hospital of Qingdao University Qingdao China

**Keywords:** acute kidney injury, circular RNA YAP1, ischaemia/reperfusion, microRNA‐21‐5p, PI3K/AKT/mTOR pathway

## Abstract

Circular RNA YAP1 (circYAP1) was reported to participate in progression of gastric cancer. However, the role of circYAP1 in acute kidney injury (AKI) remains obscure. We attempted to examine the effects of circYAP1 on ischaemia/reperfusion‐stimulated renal injury. AKI model was established by treating HK‐2 cells in ischaemia/reperfusion (I/R) environment. CircYAP1 expression in blood of AKI patients and I/R‐treated HK‐2 cells was evaluated via RT‐qPCR. CCK‐8, flow cytometry, ELISA and ROS assay were executed to test the impact of circYAP1 on cell viability, apoptosis, inflammatory cytokines and ROS generation. Bioinformatic analysis was executed to explore miRNA targets. The relativity between circYAP1 and miR‐21‐5p was verified by RT‐qPCR and luciferase assay. The functions of miR‐21‐5p in I/R‐triggered injury were reassessed. PI3K/AKT/mTOR pathway was detected by Western blot. Down‐regulated circYAP1 was observed in AKI blood samples and I/R‐treated HK‐2 cells. CircYAP1 overexpression expedited cell growth and weakened secretion of inflammatory factors and ROS generation in I/R‐disposed cells. Besides, we found circYAP1 could sponge to miR‐21‐5p. Interestingly, miR‐21‐5p overexpression overturned the repressive effects of circYAP1 on cell injury. Moreover, PI3K/AKT/mTOR pathway was activated by circYAP1 via inhibiting miR‐21‐5p. We demonstrated that circYAP1 activated PI3K/AKT/mTOR pathway and secured HK‐2 cells from I/R injury via sponging miR‐21‐5p.

## 1 INTRODUCTION

Acute kidney injury (AKI), a kind of syndrome with sudden degradation of renal function, is characterized by loss of urine creatinine.^1^ With 7.0% incidence in China, AKI becomes a representative public health matter that ranges from newborns to senior citizens.^2^ AKI is divided into pre‐kidney, intrinsic and post‐kidney, of whose pathophysiology risk factors include inflammatory diseases,^3^ traumatism^4^ and drug overdose.^5^ In addition, ischaemia/reperfusion (I/R) during kidney transplantation also is extremely likely to elicit AKI.^6^, ^7^ I/R injury, which results from the inadequate supply of oxygen and nutrients to kidney epithelial cells, manifests as acceleration of apoptosis, inflammatory response and even necrosis.^8^ Even though numerous potentially curative methods for AKI were developed, such as hemodynamic management, adequate nutrition intake and prevention of complications, obstruction of remedying AKI still remains.^9^ Renal replacement therapy (RRT) is gradually into doctors' view as clinical treatment for AKI.^10^ However, there are still disputes about the best RRT dose and therapy time.^11^ Hence, the development and generation of molecular targets will be required for further prevention and therapy of AKI.

Circular RNAs (circRNAs) are a cluster of RNA molecules with covalently bound structures that play important roles in transcription and protein translation.^12^ The most crucial function of circRNAs is to serve as sponge of microRNAs (miRNAs) or bind to RNA binding proteins and then regulate the occurrence and development of various diseases.^13^ Recent research proved that the advantageous effects of circRNAs in AKI might be linked with their abnormal expression.^14^ Up to date, the up‐expression of circRNA ciRs‐126 was confirmed in AKI patients; meanwhile, ciRs‐126 was hopefully regarded as a biomarker to predict survival rate of AKI patients.^15^ It was proved that circRNA of Yes‐associated protein 1 (circYAP1, is also named as hsa_circ_0024093), a newly discovered type of circRNA, was down‐expressed in gastric cancer tissues and could attenuate cell proliferation and invasion of gastric cancer cells.^16^ However, it is presently sealed whether circYAP1 exerts an extensive influence in I/R‐induced renal injury, and intensely little knowledge is elucidated about the regulatory mechanisms.

In this study, I/R model was established in HK‐2 cells. Consistently, the inflammatory injury of I/R‐treated HK‐2 cells was explored. We further explored the potential mechanisms of how did circYAP1 regulate the inflammatory response in I/R‐treated HK‐2 cells. This study might offer a new recognition of therapeutic target for the AKI treatment.

## 2 MATERIALS AND METHODS

### 2.1 Clinical specimens

The whole blood samples of 19 AKI cases (10 males and 9 females; age from 32 to 71 years old) and aged‐equal healthy cases (10 males and 9 females) were attained from the Affiliated Hospital of Qingdao University. None of AKI patients received any therapies. The informed consents and medical ethics certification were obtained from patients and the Medical Ethics Committee of the Affiliated Hospital of Qingdao University.

### 2.2 Cell culture

HK‐2 and HEK 293 cells were obtained from Shanghai Institutes for Biological Sciences, Chinese Academy of Sciences (Shanghai, China). All cells were maintained in DMEM medium (Gibco) which was supplemented with 10% foetal bovine serum (FBS, Gibco). For I/R treatment, HK‐2 cells were cultivated in serum‐free and glucose‐free DMEM and exposed in an incubator subchamber (Biospherix) with 1% O_2_. After 12 hours, cells were re‐cultured in normoxic condition of 21% O_2_ and DMEM with 10% FBS. In addition, cells that cultured in normoxic condition and DMEM contained 10% FBS were regarded as control group. The oxygen concentration of incubator subchamber was controlled by Compact O_2_ and CO_2_ Subchamber Controller (Biospherix).

### 2.3 Cell transfection

The overexpressing circYAP1 plasmids and siRNA of circYAP1 were colligated by Sangon. MiR‐21‐5p mimic or NC mimic was purchased from RiboBio. Cell transfection was performed by utilizing Lipofectamine 2000 Reagent (Invitrogen). After 48 hours, cells were collected with the highest transfection efficiency.

### 2.4 Reverse transcription‐quantitative Real‐time PCR (RT‐qPCR)

Total RNA was extracted by utilizing TRIzol Reagent (Invitrogen). cDNA Reverse Transcription Kit (Invitrogen) was utilized to perform reverse transcription. Next, qPCR analysis was carried out through utilizing SYBR^®^ Green PCR Kit (Qiagen) on iQ5 Real‐Time PCR amplification (Bio‐Rad). The relative expression of circYAP1 was normalized by β‐actin. U6 served as an internal control for miR‐21‐5p expression. All calculation was performed according to the $2^{-\Delta \Delta C_{t}}$ methods.

### 2.5 Cell counting kit‐8 (CCK‐8) assay

CCK‐8 reagent (Solarbio) was employed for examining cell viability. Transfected or untransfected HK‐2 cells were plated in 96‐well plates at 1 × 10^4^ cells per well. When cells reached 80% confluence, I/R treatment was carried out. After that, 10 μL of CCK‐8 reagent was added to each well and cultured at 37°C for 4 hours. The OD450 was measured by using a Microplate Reader (Pulangxin technology).

### 2.6 Flow cytometry

Guava^®^ Nexin Reagent (Luminex) was used to implement flow cytometry to test cell apoptotic potential. After cell transfection and treatment, cells were collected and suspended by DMEM. Next, 100 μL Guava Nexin solution was added into cell samples and followed by incubation for 20 minutes in dark. Finally, cell samples were detected on a Guava EasyCyte Mini System (Luminex).

### 2.7 Enzyme‐linked immunosorbent assay (ELISA)

The concentration of IL‐1β and IL‐6 in supernatants of HK‐2 cell cultures was evaluated by IL‐1β ELISA Kit (Solarbio) and IL‐6 ELISA Kit (Solarbio), respectively. Absorbance at 490 nm was measured using a Microplate Reader (Pulangxin).

### 2.8 Reactive oxygen species (ROS) assay

After transfection and treatment, cells were co‐hatched in DMEM and DCFH‐DA (final concentration for 10 μM) at 37°C for 15 minutes and followed by re‐suspending with 500 μL PBS buffer. Next, the fluorescent signals were assessed by using Olympus FV1200 Confocal microscope.

### 2.9 Dual‐luciferase reporter assay

The binding sequence of circYAP1 for miR‐21‐5p as well as mutant types was subcloned into luciferase reporter plasmid pGL3 (Promega). Then, the recombination plasmid (circYAP1^WT^ or circYAP1^MUT^) was cotransfected with miR‐21‐5p mimic or NC mimic into HEK 293 cells. After 48 hours of transfection, the luciferase reporter kit (Promega) was utilized to execute reporter assay.

### 2.10 Western blot

RIPA lysis buffer (Solarbio) supplied with PMSF (Solarbio) was used to extract total proteins from cells. Then, BCA Protein Assay Kit (Beyotime) was utilized to quantify proteins. Next, the proteins were loaded into 12% SDS‐PAGE on the Bis‐Tris Gel system (Bio‐Rad) and then were transferred to polyvinylidene fluoride (PVDF, Solarbio) membranes. Primary antibodies were added to cultivate PVDF membranes overnight at 4°C. The primary antibodies were listed following anti‐p‐PI3K (ab182651, Abcam), anti‐t‐PI3K (ab86714, Abcam), anti‐p‐AKT (ab38449, Abcam), anti‐t‐AKT (ab8805, Abcam), anti‐p‐mTOR (ab84400, Abcam), anti‐t‐mTOR (ab2732, Abcam) and anti‐β‐actin (ab179467, Abcam). After hatch with goat anti‐rabbit IgG (ab6721, Abcam) for 2 hours, the PVDF membranes were cultivated in the enhanced chemiluminescence reagent (Thermo Fisher). Eventually, the bands were tested via utilizing ImageJ software.

### 2.11 Statistical analysis

All experiments were repeated thrice. Statistical analysis was executed by using GraphPad 6.0 software. The data were presented as mean + SD *P*‐value was calculated by using Student's *t* test or ANOVA. A *P* < .05 was regarded as statistical significance.

## 3 RESULTS

### 3.1 CircYAP1 expression in blood of AKI patients and I/R‐treated HK‐2 cells

**Figure 1:**
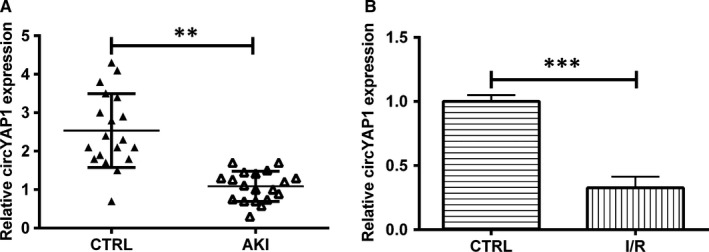
CircYAP1 expression in blood of AKI patients and I/R‐treated HK‐2 cells. A, RT‐qPCR was employed to evaluate circYAP1 expression in blood of patients with AKI (n = 19) and healthy cases (n = 19). B, HK‐2 cells were exposed in I/R environment. Then, the circYAP1 expression was detected by RT‐qPCR. **P < .01, ***P < .001

To assess evaluate difference in circYAP1 expression in whole blood of AKI patients and healthy cases, we carried out RT‐qPCR analysis. As presented in Figure 1A, AKI patients were obviously differentiating from the control, as demonstrated by the down‐regulated circYAP1 (*P* < .01). Next, HK‐2 cells were stimulated in hypoxic and glucose‐deficient environment followed by re‐oxygenation to establish AKI cell model. Figure 1B disclosed that circYAP1 was down‐expressed in I/R‐treated cells compared with control cells (*P* < .001). These results suggested that circYAP1 expression was down‐regulated in AKI patients and in HK‐2 cells after I/R treatment as well.

### 3.2 CircYAP1 moderated I/R‐induced injury of HK‐2 cells

**Figure 2:**
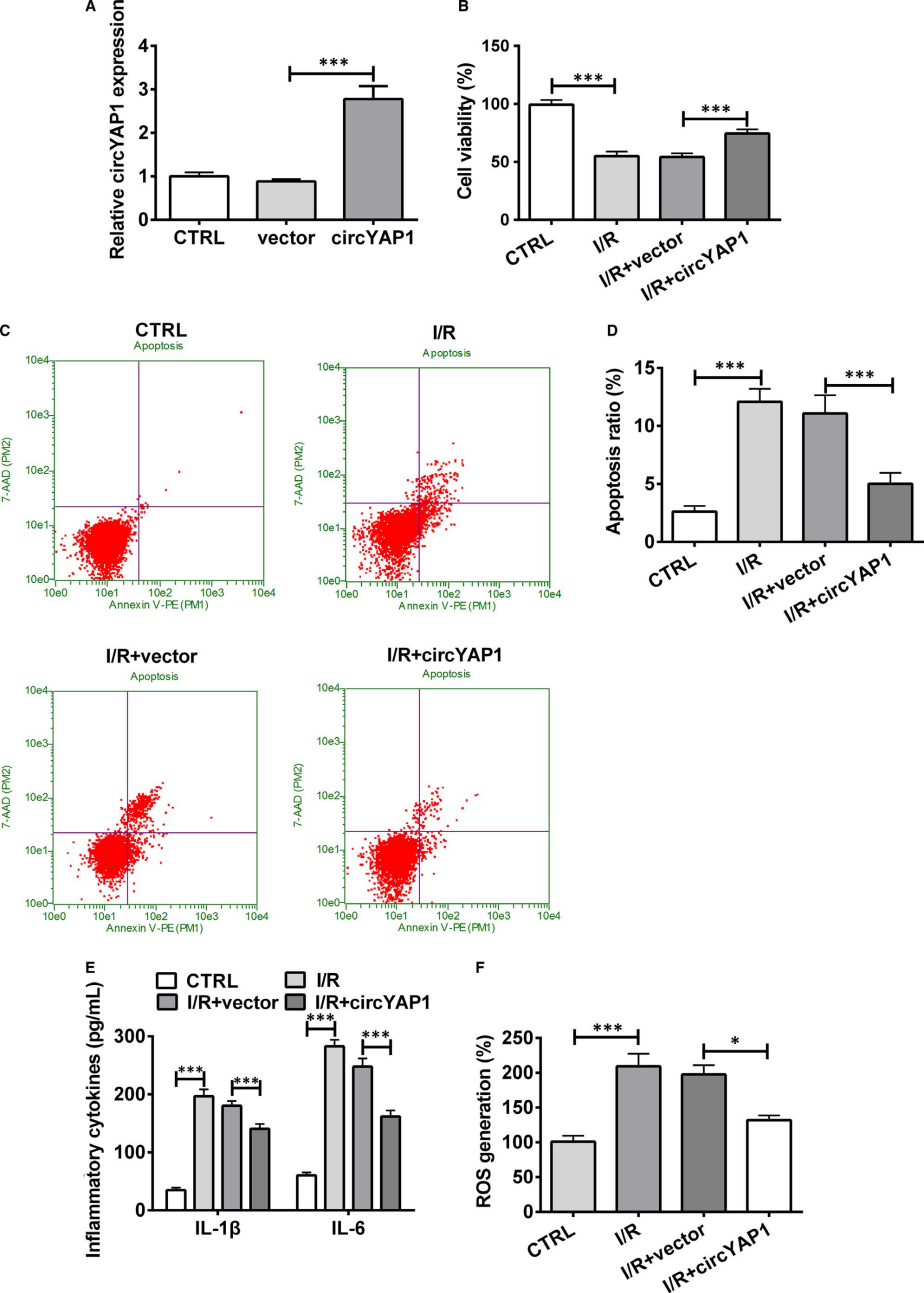
CircYAP1‐moderated I/R‐induced injury of HK‐2 cells. A, After transfection with circYAP1 overexpressing plasmid or empty vector, the transfection efficiency was assessed. HK‐2 cells were transfected with circYAP1 overexpressing plasmid or empty vector and then were stimulated in I/R environment. B, Cell viability was measured by CCK‐8 assay. C, D, Cell apoptosis ratio was assessed by flow cytometry. E, ELISA assay was utilized to examine the concentrations of inflammatory cytokines (IL‐1β and IL‐6). F, ROS generation was evaluated by ROS assay. *P < .05, ***P < .001

Considering that circYAP1 was ascertained as a disease suppressor,^16^ we asked whether circYAP1 participated in HK‐2 cell behaviours. CircYAP1 overexpressing plasmid and the corresponding vector were employed to regulate circYAP1 expression in HK‐2 cells. RT‐qPCR confirmed the overexpression of circYAP1 in circYAP1 overexpressing plasmid‐transfected group (Figure 2A; *P* < .001). After disposal in I/R condition, cell viability and apoptosis were detected by utilizing CCK‐8 and flow cytometry, respectively. In Figure 2B‐D, I/R exposure apparently reduced cell viability but facilitated cell apoptotic potential of HK‐2 cells (*P* < .001). Interestingly, the prohibition of cell growth evoked by I/R treatment was ameliorated in circYAP1 overexpressing group (*P* < .001). Meanwhile, cell inflammatory response was evaluated by detecting secretion of inflammatory cytokines and ROS generation. I/R treatment strongly upgraded the levels of IL‐1β and IL‐6, and augmented ROS generation, implying I/R‐induced inflammatory injury (Figure 2E,F; *P* < .001). Nevertheless, circYAP1 overexpression weakened the alteration of I/R treatment on inflammatory cytokines and ROS generation (*P* < .05 or *P* < .001). The above‐mentioned consequences demonstrated that circYAP1 protected HK‐2 cells against I/R‐caused injury.

### 3.3 CircYAP1 acted as a sponge for miR‐21‐5p

**Figure 3:**
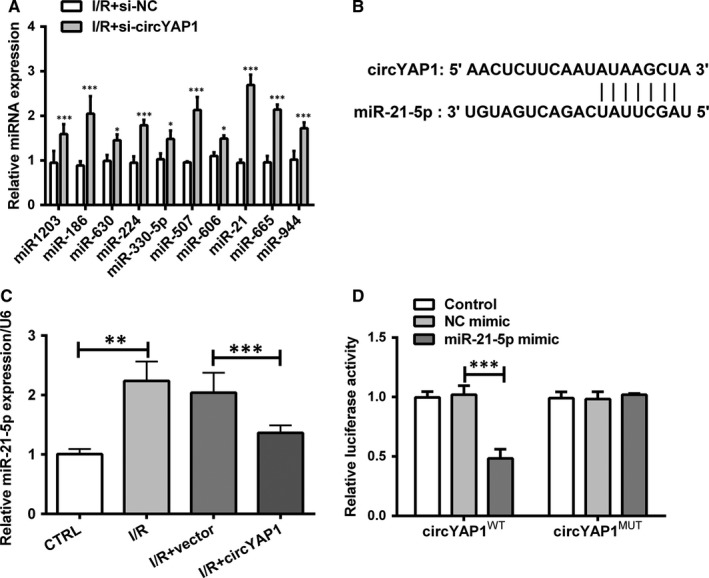
CircYAP1 acted as a sponge for miR‐21‐5p. A, HK‐2 cells were transfected with si‐circYAP1 or control (siR‐NC) and then stimulated in I/R environment. miRNA expression was assessed by RT‐qPCR. B, Schematic diagram of predicted binding sites of circYAP1 with miR‐21‐5p. C, HK‐2 cells were transfected with circYAP1 overexpressing plasmid or empty vector and then stimulated in I/R environment. The miR‐21‐5p expression was detected by RT‐qPCR. D, HEK293 cells were transfected with plasmid that contained wild‐type of circYAP1 sequence (circYAP1^WT^) or plasmid that contained mutant type of circYAP1 sequence (circYAP1^MUT^) together with miR‐21‐5p mimic or NC mimic. The relative luciferase activity was analysed by luciferase report experiment. *P < .05, **P < .01, ***P < .001

Next, bioinformatical analysis (Circular RNA Interactome, https://circinteractome.nia.nih.gov/) was employed to predict the possible miRNA targets of circYAP1. To validate the prediction, HK‐2 cells were transfected with si‐NC and si‐circYAP1. After I/R exposure, RT‐qPCR was performed to evaluate miRNA expression. As presented in Figure 3A, the expression of 10 miRNAs (miR‐1203, miR‐186, miR‐630, miR‐224, miR‐330‐5p, miR‐507, miR‐606, miR‐21‐5p, miR‐665 and miR‐944) was apparently ascended by silencing circYAP1 (*P* < .05 or *P* < .01). Among the miRNAs, the change in miR‐21‐5p expression was the most significant one. Moreover, by taking into consideration of the involvement of miR‐21‐5p in AKI,^17^ we selected miR‐21‐5p in the following research. Additionally, whether circYAP1 regulated miR‐21‐5p expression was further explored. The complimentary binding site between circYAP1 and miR‐21‐5p was shown in Figure 3B. As disclosed in Figure 3C, miR‐21‐5p expression was evidently promoted by I/R exposure (*P* < .01). Quite the opposite, the increase in miR‐21‐5p level was reversed by circYAP1 overexpressing plasmid in I/R‐treated HK‐2 cells (*P* < .001). To delve into verify the prediction of circYAP1 underlying binding to miR‐21‐5p, mutant sequence and wild‐type sequence of circYAP1 were cloned into pGL3 vector to construct circYAP1^MUT^ and circYAP1^WT^ plasmids. Then, we carried out luciferase reporter assay in HEK‐293 cells. As revealed in Figure 3D, the luciferase activity was reduced in cells that cotransfection with circYAP1^WT^ and miR‐21‐5p mimic (*P* < .001). However, there was no difference in circYAP1^MUT^ group. The aforementioned discovery gave evidence that circYAP1 acted as a sponge for miR‐21‐5p in I/R‐stimulated HK‐2 cells, hinting that miR‐21‐5p might participate in I/R‐triggered cell injury.

### 3.4 CircYAP1 relieved I/R‐caused injury through sponging miR‐21‐5p

**Figure 4:**
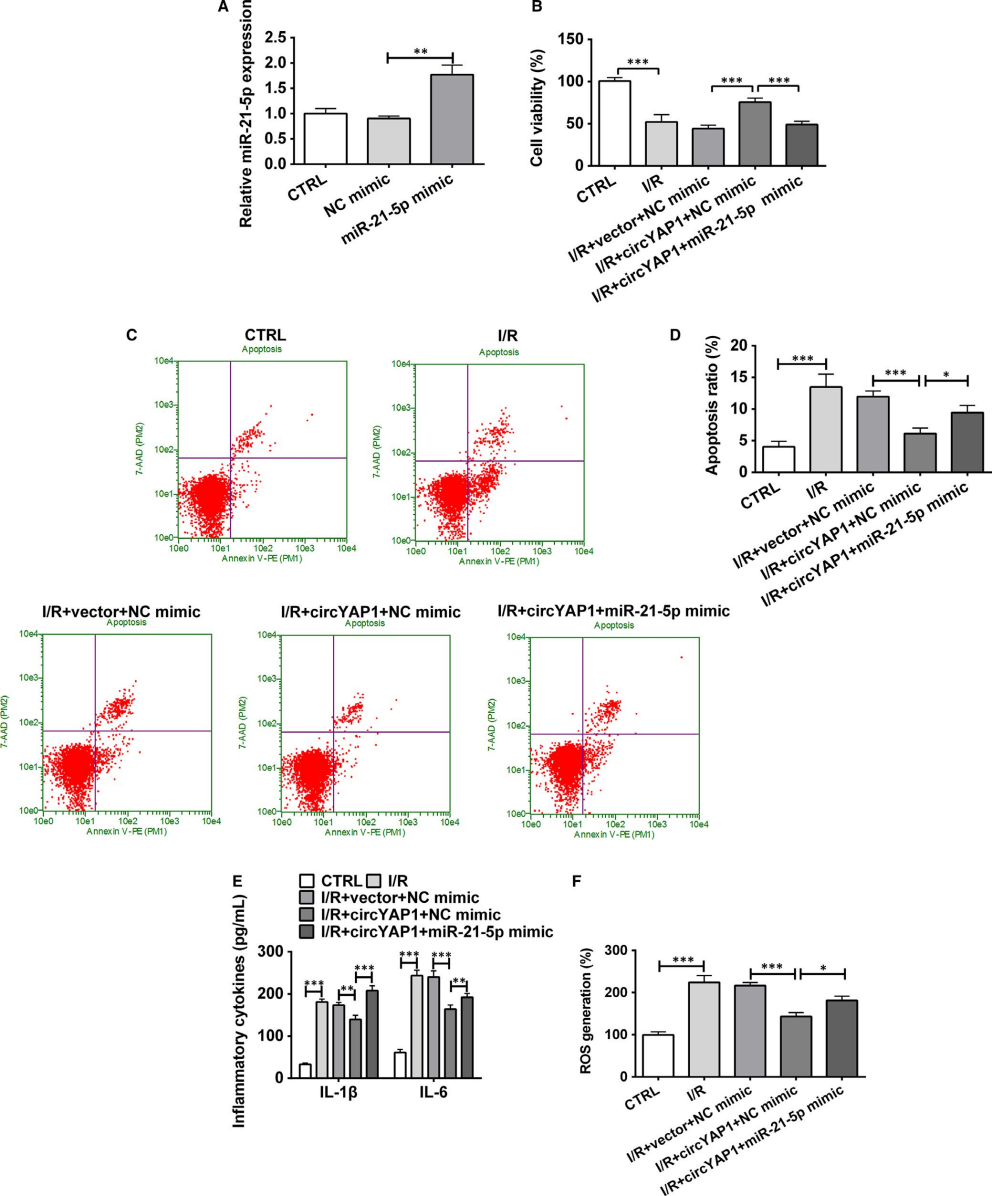
CircYAP1 relieved I/R‐caused injury through down‐regulating miR‐21‐5p. A, After transfection with miR‐21‐5p mimic or NC mimic, the transfection efficiency was assessed. After transfection with circYAP1 overexpressing plasmid (or miR‐21‐5p mimic) or the corresponding controls, HK‐2 cells were treated in I/R conditions. B, Cell viability was measured by CCK‐8 assay. C, D, Cell apoptosis ratio was assessed by flow cytometry. E, ELISA assay was utilized to examine the concentrations of inflammatory cytokines (IL‐1β and IL‐6). F, ROS generation was evaluated by ROS assay. *P < .05, **P < .01, ***P < .001

Next, miR‐21‐5p mimic was utilized to upgrade miR‐21‐5p expression in HK‐2 cells. The transfection efficiency, presented in Figure 4A, results displayed that miR‐21‐5p expression was no doubt elevated by miR‐21‐5p mimic (*P* < .01). Coinstantaneous transfection with circYAP1 overexpressing plasmid and miR‐21‐5p mimic triggered a significant reduction in cell viability (Figure 4B; *P* < .001) and an enhancement on apoptotic ratio (Figure 4C,D; *P* < .05) in I/R‐treated HK‐2 cells, by comparison with the groups that transfected with circYAP1 overexpressing plasmid and NC mimic. Consistently, Figure 4E,F revealed that miR‐21‐5p overexpression reversed the declination of inflammatory cytokines and ROS generation in circYAP1 overexpression and I/R‐treated HK‐2 cells (*P* < .05 or* P* < .01 or *P* < .001). We therefore inferred that circYAP1 might lighten I/R‐triggered injury through suppressing miR‐21‐5p expression in HK‐2 cells.

### 3.5 CircYAP1 activated PI3K/AKT/mTOR signalling pathway through sponging miR‐21‐5p in I/R‐stimulated HK‐2 cells

**Figure 5:**
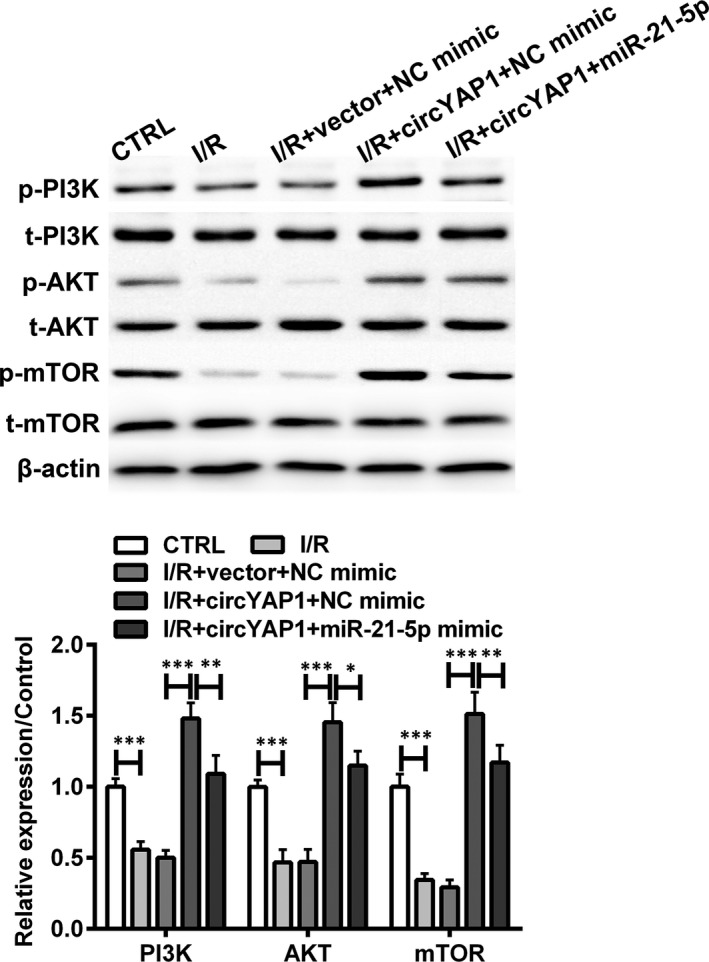
CircYAP1 activated PI3K/AKT/mTOR signalling pathway through inhibiting miR‐21‐5p expression in I/R‐stimulated HK‐2 cells. After transfection with circYAP1 overexpressing plasmid (or miR‐21‐5p mimic) or the corresponding controls, HK‐2 cells were treated in I/R conditions. The protein expression of t‐PI3K, p‐PI3K, t‐AKT, p‐AKT, t‐mTOR and p‐mTOR was tested by Western blot. The relative expression of proteins was normalized by β‐actin. *P < .05, *P < .05, ***P < .001

Considering that PI3K/AKT/mTOR signalling pathway has been extensively reported as a pivotal network in regulating the renal inflammatory response, the effect of circYAP1 on PI3K/AKT/mTOR signalling pathway was assessed to further explore the possible modulatory mechanism. I/R exposure markedly inhibited the phosphorylation of PI3K, AKT and mTOR (Figure 5; *P* < .001). By contract, protein level of p‐PI3K, p‐AKT and p‐mTOR was increased by circYAP1 overexpression in I/R exposed HK‐2 cells (*P* < .001). Beyond that, after transfection with miR‐21‐5p mimic, the above proteins were significantly allayed in I/R exposed HK‐2 cells that transfected with circYAP1 overexpressing plasmid (*P* < .01 or *P* < .001). These data indicated that PI3K/AKT/mTOR signalling pathway was potentiated by circYAP1 through restraining miR‐21‐5p expression in I/R‐stimulated HK‐2 cells.

## 4 DISCUSSION

Acute kidney injury (AKI) in kidney transplant is a widespread disease with quite a few potential causes and progressively evolves as enormous holdback for transplant management.^18^, ^19^ Here, we ascertained that circYAP1 was down‐regulated in clinical blood samples of AKI patients and I/R‐treated HK‐2 cells. CircYAP1 overexpression attenuated cell injury by expediting cell growth, suppressing inflammatory factor secretion and ROS generation in I/R‐stimulated cells. Additionally, circYAP1 triggered the declination of miR‐21‐5p expression and acted as a sponge of miR‐21‐5p. Interestingly, miR‐21‐5p overexpression overturned the repressive impacts of circYAP1 on cell injury. Moreover, PI3K/AKT/mTOR pathway was activated by circYAP1 via negatively adjusting miR‐21‐5p in I/R‐treated HK‐2 cells.

I/R‐stimulated injury is one of the major reasons which induces the onset of AKI and may be connected with high mortality.^20^ I/R injury is derived from inadequate supplement of oxygen and nutrient to organs or accumulation of metabolic waste products,^21^ and then gradually evolved into structural modification of tubular epithelial cells and immunocytes.^22^ It was demonstrated by several researchers that a multitude of inflammatory responses, including secretion of inflammatory cytokines, cell apoptosis and necrocytosis, were triggered by I/R stimulation in HK‐2 cells.^22^, ^23^ As previously reported, our research manifested that I/R exposure impeded cell viability and expedited cell apoptotic capacity of HK‐2 cells. Meanwhile, the releasing of inflammatory factor and ROS generation was elicited by I/R treatment.

CircRNAs an extraordinary sort of RNA molecules become the most worthy of exploration in the field of RNA functions.^24^ RNA sequencing data showed that a multitude of circRNAs, such as circAkt3, circPlekha7 and circMe1, were aberrantly expressed in I/R or losartan‐treated rat specimens and exerted phylactic impact in ischaemia‐induced kidney damage.^25^

Researches analysing circRNA expression in AKI have described the dysregulated expression of nearly 50 circRNAs using genome‐wide expression analysis, compared to healthy cases.^15^ Among these, the levels of hsa_circ_0045881 and hsa_circ_0001177 were obviously enhanced in whole blood of AKI patients. Additionally, an association between ciRs‐126 and curve value was disclosed.^15^ Moreover, the decreased expression of circ‐Akt3 in AKI rat model might be closely related to immune response and inflammatory damage.^25^ However, there was not any literature showed the abnormal expression of and effect of circYAP1 on AKI blood samples. It was confirmed that circYAP1 was discovered to be strongly down‐expressed in blood samples of AKI patients and I/R‐disposed HK‐2 cells. CircYAP1 is turned out during splicing process of YAP1 gene, which is an essential transcriptional factor of Hippo pathway.^16^, ^26^ Currently, only one study by Liu et al^16^ have confirmed that circYAP1 played an anti‐tumour role in gastric cancer. In this work, our data presented that circYAP1 alleviated I/R‐induced injury of HK‐2 cells by facilitating cell growth and reducing secretion of inflammatory cytokines and ROS generation.

In the past decades, a considerable amount of papers have focused on the association between circRNAs and miRNAs.^27^, ^28^ It was generally accepted that circRNAs could serve as sponges of miRNAs and accordingly weakened miRNA activity.^29^ For instance, Yang et al^30^ stated that silencing circ_008018 ameliorated I/R‐induced injury of cerebrum cells by sponging miR‐99a. CircInteractome, a burgeoning online analysis tool, is applied extensively in predicting miRNA‐circRNA interactions.^31^, ^32^ In this study, we demonstrated that there were 10 potential miRNAs targeted circYAP1 by employing CircInteractome and RT‐qPCR.

Furthermore, there was evidence regarding the importance in AKI process of miRNAs. Previous studies utilizing Dicer‐knockout mice model resulted in the observation of crucial dysregulated miRNA molecules, including miR‐486, miR‐207 and miR‐455‐3p, suggesting pathogenical or protective roles for miRNA expression in ischaemic AKI.^33^, ^34^ For instance, miR‐192 expression in blood plasma was perhaps useful to diagnose patients with AKI and recovery procedure of renal damage.^35^ In the context of clinical and experiment data, miR‐709 has been associated with regulatory mechanism by which mitochondrial dysfunction and proximal tubular cell apoptosis were ameliorated.^36^ In contrast, another AKI‐related miRNA (miR‐688) acted protective role in AKI by inhibiting mitochondrial fragmentation.^37^ Previous researches indicated that miR‐21 was up‐regulated both in kidney tissues and in urine of AKI patients.^38^, ^39^ Additionally, in vitro study revealed that miR‐21 restrained cell growth and autophagy of I/R‐injured rat renal tubular epithelial cells.^40^ But few reports about the upstream regulator of miR‐21 in AKI have been conducted. In addition, no common effect of miR‐21 has been identified in previous studies, reflecting the shortage of well‐investigated reports in the field of AKI process. In our experiments, we discovered that miR‐21‐5p expression was obviously aggrandized by I/R treatment while declined by circYAP1 in HK‐2 cells. Additionally, it was proved that circYAP1 served as a sponge for miR‐21‐5p by luciferase assay. Furthermore, the catabatic influences of circYAP1 in I/R injury were reversed by miR‐21‐5p up‐regulation. All these results hinted that circYAP1 might impede I/R injury of HK‐2 cells via sponging miR‐21‐5p.

It was demonstrated that PI3K/AKT/mTOR pathway contributed to processes of several inflammatory diseases, such as osteoarthritis^41^ and intestinal colitis.^42^ PI3K/AKT/mTOR signalling pathway is also critical apoptosis‐related pathway which is usually interrupted under hypoxia condition.^43^ For example, accumulating evidence further demonstrated that PI3K/AKT/mTOR pathway was potentiated to protect rat from I/R‐stimulated dysmnesia.^44^ Interestingly, pharmaceutics research demonstrated that PI3K/AKT/mTOR pathway was involved in cell apoptotic potential and inflammatory responses of renal I/R model, and activation of pathway‐associated proteins was an indispensable step in this process.^45^ The PI3K/AKT/mTOR pathway is also regarded as an amenable pathway to acute renal injury.^46^ Specifically, the protective effect of tempol on I/R‐evoked renal injury was associated with the activation of PI3K/AKT/mTOR pathway. In addition, PI3K/AKT/mTOR signalling pathway was involved in the inflammatory response of kidney tubular epithelial cells.^47^ Moreover, previous investigation of I/R injury presented significant reduction in inflammatory cytokine (TNF‐α and IL‐1β) concentration in mmu_circRNA_005186‐silenced cells, which process might be closely related to mTOR protein.^48^ In our research, we first linked circYAP1 regulation to PI3K/AKT/mTOR pathway in I/R‐injured HK‐2 cells. In addition, this process was mediated by down‐regulation of miR‐21‐5p.

In general, the study indicated that circYAP1 secured HK‐2 cells from I/R‐induced injury via abating miR‐21‐5p expression, implying the suppressive effects of circYAP1 on AKI. Advances in knowledge of circYAP1‐mediated molecular mechanisms involved in the I/R‐induced injury of HK‐2 cells might shed light on the development of effective therapies for AKI. In future, in vivo experiments will be a crucial key for our further research.

## CONFLICT OF INTEREST

The authors declare that there are no conflicts of interest.

## AUTHOR CONTRIBUTION

TH, YC and ZD provided theoretical guidance and designed experiments. TH, YC, HW and QW performed experiments and analysed results. TH, YC, JJ, XS and ZD interpreted data and drafted the manuscript. All authors revised the manuscript and contributed substantially to this research.

## Data Availability

The data used to support our findings are available from the corresponding author on reasonable request.

## References

[1] Connell A , Laing C . Acute kidney injury. Clin Med. 2015;15:581‐584. 10.7861/clinmedicine.15-6-581 PMC4953266 26621953

[2] Yang L , Xing G , Wang L , et al. Acute kidney injury in China: a cross‐sectional survey. Lancet. 2015;386:1465‐1471. 26466051 10.1016/S0140-6736(15)00344-X

[3] Koza Y . Acute kidney injury: current concepts and new insights. J Inj Violence Res. 2016;8:58‐62. 26804946 10.5249/jivr.v8i1.610 PMC4729334

[4] Harrois A , Libert N , Duranteau J . Acute kidney injury in trauma patients. Curr Opin Crit Care. 2017;23:447‐456. 29035925 10.1097/MCC.0000000000000463

[5] Naughton CA . Drug‐induced nephrotoxicity. Am Fam Physician. 2008;78:743‐750. 18819242

[6] Zuk A , Bonventre JV . Acute kidney injury. Ann Rev Med. 2016;67:293‐307. 26768243 10.1146/annurev-med-050214-013407 PMC4845743

[7] Thadhani R , Pascual M , Bonventre JV . Acute renal failure. N Engl J Med. 1996;334:1448‐1460. 8618585 10.1056/NEJM199605303342207

[8] Zhu H , Sun A . Programmed necrosis in heart disease: molecular mechanisms and clinical implications. J Mol Cell Cardiol. 2018;116:125‐134. 29426003 10.1016/j.yjmcc.2018.01.018

[9] Rahman M , Shad F , Smith MC . Acute kidney injury: a guide to diagnosis and management. Am Fam Physician. 2012;86:631‐639. 23062091

[10] Villa G , Ricci Z , Ronco C . Renal replacement therapy. Crit Care Clin. 2015;31:839‐848. 26410148 10.1016/j.ccc.2015.06.015

[11] Lines S , Lewington A . Acute kidney injury. Clin Med. 2009;9:273‐277. 10.7861/clinmedicine.9-3-273 PMC4953621 19634397

[12] Vicens Q , Westhof E . Biogenesis of circular RNAs. Cell. 2014;159:13‐14. 25259915 10.1016/j.cell.2014.09.005

[13] Wang Y , Mo Y , Gong Z , et al. Circular RNAs in human cancer. Mol Cancer. 2017;16:25. 28143578 10.1186/s12943-017-0598-7 PMC5282898

[14] Haddad G , Kolling M , Lorenzen JM . The hypoxic kidney: pathogenesis and noncoding RNA‐based therapeutic strategies. Swiss Med Wkly. 2019;149:w14703. 30636273 10.4414/smw.2019.14703

[15] Kölling M , Seeger H , Haddad G , et al. The circular RNA ciRs‐126 predicts survival in critically Ill patients with acute kidney injury. Kidney Int Rep. 2018;3:1144‐1152. 30197981 10.1016/j.ekir.2018.05.012 PMC6127441

[16] Liu H , Liu Y , Bian Z , et al. Circular RNA YAP1 inhibits the proliferation and invasion of gastric cancer cells by regulating the miR‐367‐5p/p27 (Kip1) axis. Mol Cancer. 2018;17:151. 30336780 10.1186/s12943-018-0902-1 PMC6193296

[17] Pavkovic M , Robinson‐Cohen C , Chua AS , et al. Detection of drug‐induced acute kidney injury in humans using urinary KIM‐1, miR‐21, ‐200c, and ‐423. Toxicol Sci. 2016;152:205‐213. 27122240 10.1093/toxsci/kfw077 PMC5009468

[18] Dudreuilh C , Aguiar R , Ostermann M . Acute kidney injury in kidney transplant patients. Acute Med. 2018;17:31‐35. 29589603

[19] Bellomo R , Kellum JA , Ronco C . Acute kidney injury. Lancet. 2012;380:756‐766. 22617274 10.1016/S0140-6736(11)61454-2

[20] Ostermann M , Liu K . Pathophysiology of AKI. Best Pract Res Clin Anaesthesiol. 2017;31:305‐314. 29248138 10.1016/j.bpa.2017.09.001

[21] Le Dorze M , Legrand M , Payen D , Ince C . The role of the microcirculation in acute kidney injury. Curr Opin Crit Care. 2009;15:503‐508. 19829106 10.1097/MCC.0b013e328332f6cf

[22] Kezić A , Stajic N , Thaiss F . Innate immune response in kidney ischemia/reperfusion injury: potential target for therapy. J Immunol Res. 2017;2017:6305439. 28676864 10.1155/2017/6305439 PMC5476886

[23] Masola V , Zaza G , Bellin G , et al. Heparanase regulates the M1 polarization of renal macrophages and their crosstalk with renal epithelial tubular cells after ischemia/reperfusion injury. FASEB J. 2018;32:742‐756. 28970256 10.1096/fj.201700597R

[24] Hsiao KY , Sun HS , Tsai SJ . Circular RNA – new member of noncoding RNA with novel functions. Exp Biol Med. 2017;242:1136‐1141. 10.1177/1535370217708978 PMC5478007 28485684

[25] Fang M , Liu S , Zhou Y , et al. Circular RNA involved in the protective effect of losartan on ischemia and reperfusion induced acute kidney injury in rat model. Am J Transl Res. 2019;11:1129‐1144. 30899412 PMC6413261

[26] Stein C , Bardet AF , Roma G , et al. YAP1 exerts its transcriptional control via TEAD‐mediated activation of enhancers. PLoS Genet. 2015;11:e1005465. 26295846 10.1371/journal.pgen.1005465 PMC4546604

[27] Ebbesen KK , Hansen TB , Kjems J . Insights into circular RNA biology. RNA Biol. 2017;14:1035‐1045. 27982727 10.1080/15476286.2016.1271524 PMC5680708

[28] Kulcheski FR , Christoff AP , Margis R . Circular RNAs are miRNA sponges and can be used as a new class of biomarker. J Biotechnol. 2016;238:42‐51. 27671698 10.1016/j.jbiotec.2016.09.011

[29] Hansen TB , Jensen TI , Clausen BH , et al. Natural RNA circles function as efficient microRNA sponges. Nature. 2013;495:384‐388. 23446346 10.1038/nature11993

[30] Yang X , Ji H , Yao Y , et al. Downregulation of circ_008018 protects against cerebral ischemia‐reperfusion injury by targeting miR‐99a. Biochem Biophys Res Commun. 2018;499:758‐764. 29605297 10.1016/j.bbrc.2018.03.218

[31] Dudekula DB , Panda AC , Grammatikakis I , De S , Abdelmohsen K , Gorospe M . CircInteractome: a web tool for exploring circular RNAs and their interacting proteins and microRNAs. RNA Biol. 2016;13:34‐42. 26669964 10.1080/15476286.2015.1128065 PMC4829301

[32] Panda AC , Dudekula DB , Abdelmohsen K , Gorospe M . Analysis of circular RNAs using the web tool circinteractome. Methods Mol Biol. 2018;1724:43‐56. 29322439 10.1007/978-1-4939-7562-4_4 PMC5897125

[33] Fan P‐C , Chen C‐C , Chen Y‐C , Chang Y‐S , Chu P‐H . MicroRNAs in acute kidney injury. Hum Genomics. 2016;10:29. 27608623 10.1186/s40246-016-0085-z PMC5016954

[34] Wei Q , Bhatt K , He H‐Z , Mi Q‐S , Haase VH , Dong Z . Targeted deletion of Dicer from proximal tubules protects against renal ischemia‐reperfusion injury. J Am Soc Nephrol. 2010;21:756‐761. 20360310 10.1681/ASN.2009070718 PMC2865746

[35] Zhang L , Xu Y , Xue S , et al. Implications of dynamic changes in miR‐192 expression in ischemic acute kidney injury. Int Urol Nephrol. 2017;49:541‐550. 28035621 10.1007/s11255-016-1485-7 PMC5321705

[36] Guo Y , Ni J , Chen S , et al. MicroRNA‐709 mediates acute tubular injury through effects on mitochondrial function. J Am Soc Nephrol. 2018;29:449‐461. 29042455 10.1681/ASN.2017040381 PMC5791060

[37] Chun N , Coca SG , He JC . A protective role for microRNA‐688 in acute kidney injury. J Clin Invest. 2018;128:5216‐5218. 30418172 10.1172/JCI124923 PMC6264641

[38] Ramachandran K , Saikumar J , Bijol V , et al. Human miRNome profiling identifies microRNAs differentially present in the urine after kidney injury. Clin Chem. 2013;59:1742‐1752. 24153252 10.1373/clinchem.2013.210245 PMC3870155

[39] Saikumar J , Hoffmann D , Kim TM , et al. Expression, circulation, and excretion profile of microRNA‐21, ‐155, and ‐18a following acute kidney injury. Toxicol Sci. 2012;129:256‐267. 22705808 10.1093/toxsci/kfs210 PMC3499041

[40] Liu X , Hong Q , Wang Z , Yu Y , Zou X , Xu L . MiR‐21 inhibits autophagy by targeting Rab11a in renal ischemia/reperfusion. Exp Cell Res. 2015;338:64‐69. 26302266 10.1016/j.yexcr.2015.08.010

[41] Xue J‐F , Shi Z‐M , Zou J , Li X‐L . Inhibition of PI3K/AKT/mTOR signaling pathway promotes autophagy of articular chondrocytes and attenuates inflammatory response in rats with osteoarthritis. Biomed Pharmacother. 2017;89:1252‐1261. 28320092 10.1016/j.biopha.2017.01.130

[42] Kim H , Banerjee N , Barnes RC , et al. Mango polyphenolics reduce inflammation in intestinal colitis‐involvement of the miR‐126/PI3K/AKT/mTOR axis in vitro and in vivo. Mol Carcinog. 2017;56:197‐207. 27061150 10.1002/mc.22484 PMC5053910

[43] Chang L , Graham PH , Hao J , et al. PI3K/Akt/mTOR pathway inhibitors enhance radiosensitivity in radioresistant prostate cancer cells through inducing apoptosis, reducing autophagy, suppressing NHEJ and HR repair pathways. Cell Death Dis. 2014;5:e1437. 25275598 10.1038/cddis.2014.415 PMC4237243

[44] Amini‐Khoei H , Saghaei E , Mobini GR , et al. Possible involvement of PI3K/AKT/mTOR signaling pathway in the protective effect of selegiline (deprenyl) against memory impairment following ischemia reperfusion in rat. Neuropeptides. 2019;77:101942. 31272684 10.1016/j.npep.2019.101942

[45] Wei Q , Zhao J , Zhou X , Yu L , Liu Z , Chang Y . Propofol can suppress renal ischemia‐reperfusion injury through the activation of PI3K/AKT/mTOR signal pathway. Gene. 2019;708:14‐20. 31082504 10.1016/j.gene.2019.05.023

[46] Ferro A , Morais S , Rota M , et al. Tobacco smoking and gastric cancer: meta‐analyses of published data versus pooled analyses of individual participant data (StoP Project). Eur J Cancer Prev. 2018;27:197‐204. 29595756 10.1097/CEJ.0000000000000401

[47] Du C , Zhang T , Xiao X , Shi Y , Duan H , Ren Y . Protease‐activated receptor‐2 promotes kidney tubular epithelial inflammation by inhibiting autophagy via the PI3K/Akt/mTOR signalling pathway. Biochem J. 2017;474:2733‐2747. 28694352 10.1042/BCJ20170272

[48] Zhang P , Ming Y , Ye Q , Niu Y . Comprehensive circRNA expression profile during ischemic postconditioning attenuating hepatic ischemia/reperfusion injury. Sci Rep. 2019;9:264. 30670716 10.1038/s41598-018-36443-8 PMC6342922

